# Jumping Wave Characteristic during Low Plasticity Burnishing Process

**DOI:** 10.3390/ma14061441

**Published:** 2021-03-16

**Authors:** Stefan Dzionk, Michal Dobrzynski, Bogdan Ścibiorski

**Affiliations:** Institute of Production Engineering and Materials, Faculty of Mechanical Engineering and Ship Technology, Gdansk University of Technology, 80-233 Gdansk, Poland; michal.dobrzynski@pg.edu.pl (M.D.); bogdan.scibiorski@pg.edu.pl (B.Ś.)

**Keywords:** jumping wave, surface layer, low plasticity burnishing process

## Abstract

During the low plasticity burnishing process of soft materials such as carbon steel with a hardness of up to 40 HRC (Rockwell grade) a raised structure of the material known as the Jumping Wave forms in front of the tool roll. This phenomenon significantly disturbs the burnishing process, but is very poorly described in the literature. This article presents studies of this phenomenon on the example of burnished 1.0562 steel. The research concerns the changes in the surface structure of the processed material as well as changes in the structure of the material during this process. The research shows changes in the geometric structure of the surface made in the 3D system and their parametric description. Moreover, the work presents an analysis of the metallographic structure in the tool zone. The research showed occurrence of material slippages in the wave in front of the tool, which creates an additional structure on the surface. These tests make it possible to better understand the process of changes that take place in the surface layer of the processed element in the low plasticity burnishing process.

## 1. Introduction

The burnishing process is a surface finishing method of the plastic deformation of the surface in order to obtain high accuracy and small surface roughness parameter (Ra = 0.08 µm) [1]. In this process there is no removal of material from the surface and no significant consumption of tools and auxiliary materials, and so therefore the process is environmentally friendly. The surface is deformed with special and hard tools, such as balls, discs, rollers and is called rolling burnishing. Sliding burnishing uses tools which are made of very hard materials and the burnishing surfaces of these tools are shaped as a paraboloid, sphere, etc. During burnishing of soft materials, of which their hardness is below 40 HRC, an additional material structure is created in front of the tool, which in the literature [1] and in workshop slang is referred as “jumping wave”. The formation of such a wave greatly destabilizes the burnishing process. The main problem is the wave size increase during the burnishing of long elements. The increasing wave destabilizes the burnishing parameters. The increasing amount of material that is premixed by the burnishing tool causes a rapid increase in burnishing force, processing components and tool load. The enhanced burnishing force increases the pressures and loading of the machine tool system and affects the structure of the surface layer during this process. Another problem is the pulling in of the resulting wave under the burnishing tools. Being pulled in a wave under the tools damages the machined surface. In the literature, the phenomenon of a wave forming in front of the tool was noticed in the 1950s [1]. This problem has not been investigated and described; however, solutions limiting the impact of this wave on the stability of machining have been proposed, e.g., by using an additional cutting tool which reduces the wave size. The knowledge of the phenomena causing the creation and movement of the wave will enable sophisticated control of the burnishing process and accurate results in the creating of the surface layer of the processed component.

### 1.1. Literature Review

The problem of the burnishing of the soft materials in the literature has been extensively described for both steel [1,2,3,4,5,6,7,8,9,10,11,12,13,14,15,16,17,18,19,20] and other materials [21,22,23,24,25,26,27,28,29,30,31,32,33]. This applied both rolling [3,4,6] and sliding [2,10,14] burnishing. The majority of these articles [1,2,3,4,5,6,7,8,9,10,11,12,13,14,15,16,17,18,19,20,21,22,23,24,25,26,27,28,29,30,31,32,33,34] present studies that link the input parameters with the properties of the obtained surface layer. Many of these articles describe the obtained properties of the surface layer in relation to the geometric surface structure, structure and micro-hardness of the surface layer and the stresses present in it, as well as functional properties such as fatigue strength, surface friction and wear, corrosion resistance, etc. In these articles, the burnishing process is described according to a simple scheme and the problem of the occurrences of the wave before the tool is often missed. On the other hand, the obtained surface layer structures are very well described in the form of post-processing surface profilograms, microphotographs of metallographic structures of surface defects and other functional features. In the presented articles can be found research results that are not sufficiently explained based on the methods used. For example, the article [29] noted that the material from the surface as a result of burnishing was pressed into the surface layer of the part thus the authors explain it by the displacement of the material as a result of shear instability, while it is not excluded that this may be the result of the phenomena that occur in the wave before the tool.

In review publications on the subject of the burnishing process [1,35,36,37], the phenomenon occurring in this process is presented only in general terms, while there is no detail description of the phenomena occurring during wave formation.

In the next group of articles [38,39] is presented the deformation of the irregularities by mathematical analytical models. These models generally try to map the local interaction of the tool on the material, but usually concern only the static interaction. These calculations do not cover a larger research area and the phenomenon of wave formation is not taken into account.

In terms of modeling, numerical models are a large group [34,35,36,37,40,41,42,43]. Contact interactions between material and tool are usually modeled in these publications. Local deformations of inequalities [42] are also modeled in the literature, whereas modeling in a larger area of influence is rare and they do not explain the phenomena of wave generation.

Some phenomena of wave formation in front of the tool were presented in the article on wear processes [44]. The description of the phenomenon presented there does not have an exact replica in the process of burnishing because the tools with edges are used in this process. These tools cause the processes presented there to correspond more to the processes occurring during cutting with tools of a negative rake angle. However, in the behaviour of the workpiece material, certain analogies can be observed which also occur during the burnishing process. In particular, there are micro cracks on the surface, crack-like features, tears, etc.

On the basis of the presented literature review, it can be concluded that the phenomena occurring during burnishing, and especially the wave formation, is not sufficiently described in the literature. This is an important subject as such a phenomenon is common in low plasticity burnishing process. A more accurate explanation of the wave creating phenomenon will enable better planning of the burnishing process and avoidance of the formation of flaws on the surfaces, and in particular the surface flaking.

### 1.2. Process of Burnishing and Surface Layer Structure

**Figure 1:**
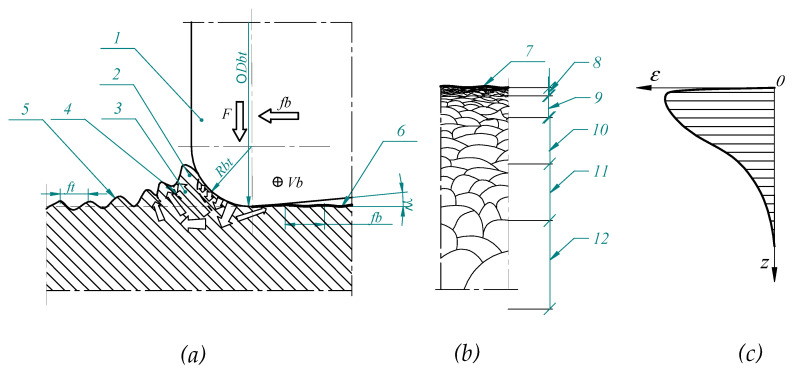
Smoothing and strengthening rolling burnishing: (**a**)—scheme of process, (**b**)—structure of surface layer after burnishing (**c**)—graph of deformation in the surface layer after burnishing process, 1—burnishing roller, 2—structure of material before burnishing tool (jumping wave), 3—schematic presentation of displacement of material grains, 4—slipping surface of burnishing material 5—surface before burnishing, 6—surface after burnishing, 7—zone of geometric structure of surface, 8—zone of significant grain deformation, 9—zone of predominated of the grain deformation, 10—zone of plastic and elastic surface deformation, 11—zone of elastic deformation, 12—the core of material, *V_b_*—burnishing speed, *f_b_*—burnishing feed rate, *F*—burnishing force, *R_bt_—*rounding radius of burnishing tool, *D_bt_*—diameter of burnishing tool, *γ*—angle of surface smoothing.

Figure 1a shows the scheme of rolling burnishing process. The process is carried out by means of a roller whose geometrical shape is described by parameters: diameter (*D_bt_*), rounding radius (*R_bt_*) and the angle of surface waviness suppression (*γ*). A tool of this type is usually made of hardened tool steel or cemented carbide. The surface used to suppress the waviness is in the shape of a truncated cone with an apex angle of 2*γ*. It is designed to limit the outflow of burnished material behind the tool. This Figure shows a schematic representation of the wave that is created in front of the burnishing tool. The wave is caused by the influence of the tool on the material whereby the material is pushed out from the deeper layers of the processing part and then pressed back in again by the tool. This phenomenon causes a partial orientation of the material grains and in the near-surface area these grains are also strongly deformed. The rising of the material in front of the tool generates significant shear stresses, which results in material slippage and in the creation of additional surface irregularities on the wave front. These phenomena are schematically shown as items 3 and 4 in Figure 1.

Figure 1b shows schematically the structure of the surface layer that is obtained in the burnishing process. The surface layer has different properties at different depths and the literature [1] uses the division of the zones diversified in terms: hardness, internal stresses and material structures. The first zone (item 7 in the Figure 1) on the component surface is the roughness zone includes the scope of irregularities of the surface created as a result of processing. In the cold plastic deformation process are reduced the structures of irregularities, mainly their shapes and height. Under the roughness zone is occurred zone which is characterized as a material grain fragmentation area. The material grains in this zone are crushed and moved along surface (item 8 in the Figure 1). This zone is characterized by the high hardness and high compressive stresses. This zone is often divided into additional sub-zones depending on the level grain fragmentation (item 9 in the Figure 1). During planning of the manufacturing process, it should be taken into consideration that too much deformation range and movement of the material in this zone may cause the formation of the discontinuities and micro cracks, which may cause surface flaws. The next zone (item 10 in the Figure 1) is a plastic deformation zone. In this zone the material grains are not fragmented but are deformed only plastic and they have obtained an elongation shape. This zone is characterized by slight material strengthening, increase hardness and also compressive stress. Processing parameters, including the burnishing force, determine the depth of changes in the surface layer of the processing material and also impact on the thickness of particular zones. The deepest zone referred to is the elastic deformation zone (item 11 in the Figure 1). Material in this zone is only elastically deformed by the stresses occurring there. Part c in Figure 1 shows schematically the range of displacements in the top layer structure resulting from the burnishing process.

## 2. Materials and Methods

Samples were made of steel 1.0562 in the form of shaft with a diameter *ɸ* 97 mm and length *l* = 350 mm. The material data of the samples are presented in Table 1. The chemical composition of the used steel has been confirmed by spectral testing carried out on the equipment made by SPECTRO. This steel was delivered by CELSA GROUP. The samples were put in a three jaws self-centering chuck with the support of the rotary center. After turning, the cylindrical form of the obtained surface was checked by means of a caliper with accuracy of 0.01 mm. During testing of the diameter the measurement deviation did not exceed ±0.02 mm along the entire length of the shaft. The burnishing tool was set in a tool post in the plane of the axis of rotation of the shaft. The parameters of the working element of the burnishing tool were: diameter of burnishing tool *D_bt_* = 40 mm, rounding radius *R_bt_* = 2.5 mm, angle of surface smoothing *γ* = 5 deg. The working element was made of tool steel with a hardness 65 ± 2 HRC and its working surface roughness was Ra = 0.08 µm. Table 2 presents the parameters of the burnishing process. The machine oil type L-AN 46 (ISO 3448 [45]) was used during the burnishing process. In order to study the wave phenomenon, the process was stopped and the structures formed before tool on the shaft were studied.

Samples for metallographic tests in the dimension 10 mm × 10 mm × 10 mm were cut from the shaft and polished next etched (Nital reagent 3%) by about 15 s in order to make the structure visible. A metallographic microscope type OLYMPUS BX51 with software OLYMPUS Stream Motion was applied to sample testing. The surface topography measurements of the processed samples were made using 3D Optical Profiler S neox (Sensofar 2019, Sensofar Metrology, Terrassa, Spain) [46] with objective 10× EPI (for turned sample) and 5× EPI (for burnished sample) of Nikon. The system was controlled by SensoSCAN 6.6 software (v.6.6, Sensofar Metrology, Terrassa, Spain), and the basic details of the measurement were set as follows:

- Topography: 1354 px × 1018 px;
- Pixel size: 1.3 μm/pixel (for turned sample) and 2.6 μm/pixel (for burnished sample);
- Axis Z-Scan step of 2 μm (for turned sample) and 12 μm (for burnished sample);
- Threshold 3%;
- Algorithm: Confocal Fusion.

Surface analyses were performed using the MountainsMap 7.1 software package (2019, Digital Surf Besançon, France; made available Optotom, Warsaw, Poland). In order to analyse surface texture for turned sample the measured surface has been approximated using polynomials of the power of 2 (F-Operator). For the burnished part the general slope of a sample using levelling process was removed. Therefore, the “Level” Operator was applied, in accordance with ISO 25178 [47], that was based on the least-squares form fitting such as a levelling using an LS-plane.

## 3. Results and Discussion

**Figure 2:**
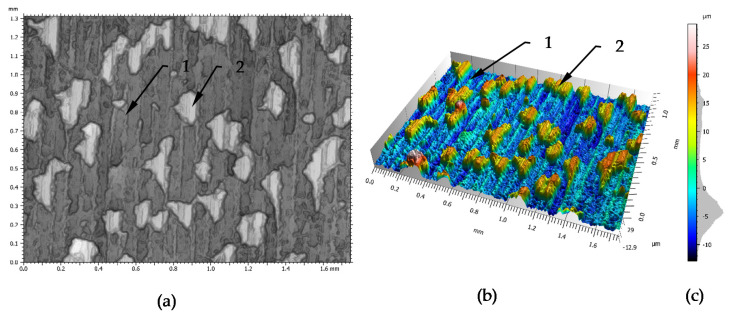
Surface structure after turning: (**a**)—photograph of surface, (**b**)—3-D surface scanning, (**c**)—scale of height and the ordinate histogram, 1—turning tool trace, 2—hard material grains on the surface. The surface topography measurements were made with objective 10× EPI and Z-Scan step of 2 μm.

Research of the wave formation process in front of the burnishing tool were performed on steel specimens (material data—Table 1) that had been turned before burnishing (processing data—Table 2). Figure 2 shows the surface structure of the samples after turning, which was measured by a confocal microscope. In this Figure are visible traces (1) of the cutting tool and particles of protruding above the surface (2) of the processed material.

The surface structure seen in Figure 2 was measured and described by the roughness surface parameters. The results of this geometric structure made according to ISO 25178 [47] are presented in Table 3. Based on the 3-D view (Figure 2) and a proportion of Sp to Sv parameter (Table 3) it can be confirmed the occurrence of single hills of the geometric structure of the surface. The maximum height (Sz) value was 41.8 μm and the plateaus were predominant and maximum peak height (Sp) represented it to almost 70% of maximum height (Sz). Presented skewness parameter (Ssk) was used to evaluate deviations in the height distribution. Positive skewness (Ssk = 1.12) indicates the presence of high plateaus above a smoother valley. The kurtosis (Sku = 3.09) value was slightly higher than 3, which indicates the presence of some sharp valleys in the texture. The root mean square height (Sq) value was about 25% higher than arithmetical mean height (Sa) value. Such differences are characteristic for irregular random profile, where individual maximum and minimum height values, observed in the profile, have greater effect on Sq than on Sa.

**Figure 3:**
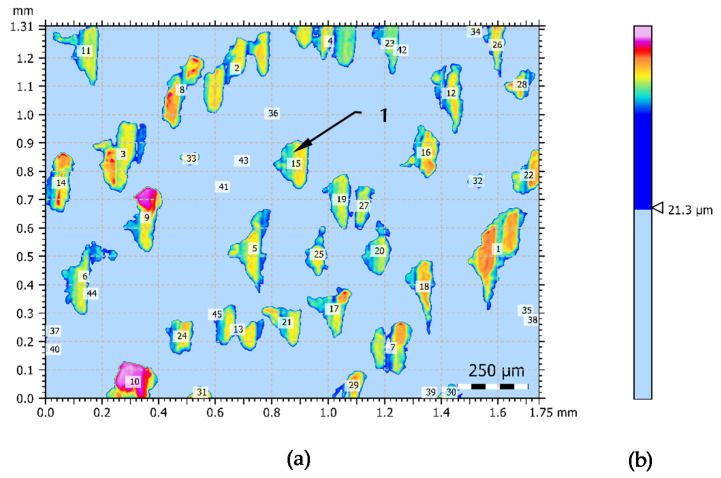
Island analysis of the turning surface: (**a**)—view of island, (**b**)—cut-off value of threshold of the island across a 0–1.8 µm scale, 1—island on the surface.

Due to the dominance of peaks/plateaus in the surface structure, additional analyses have been made in purpose of more precise description of this structure. On the basis of the data in Table 3 it was assumed that the lack of individual islands in the structure presented in Figure 2 would reduce this structure to a height of about 20 µm. The cut-off threshold is assumed to be 21.3 µm. The outline of the islands surface structure and the magnitude of the threshold is presented in Figure 3. In this Figure may be seen that a few large elements of the peaks reach a considerable height.

Peaks that change the symmetry of the surface structure were characterized by additional parameters. The parameters which describe the structure visible in the Figure 3 are presented in Table 4.

**Figure 4:**
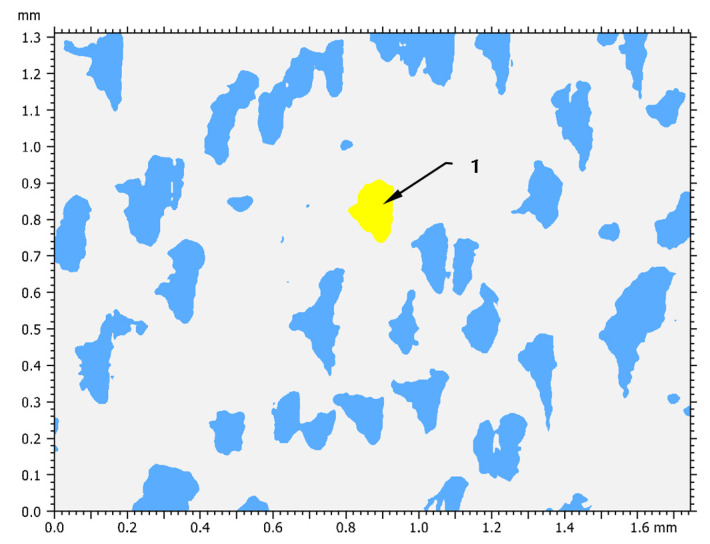
Grains analysis of the turning surface: 1—exemplary grain.

The grains presented in Figure 2 and Figure 4 represent a harder structure of the material and are an important element in the burnishing process. Therefore, an additional analysis of surface granularity based on the outlines of this structure was performed.

Table 5 shows the general grains data of the surface structure. This analysis was carried out on the basis of the binary form of the grain shape.

The results of a more detailed statistical analysis of the grain shape form are presented in Table 6. Based on the above-mentioned results, it is possible to notice that the shape of the grains is very irregular and dimensionally varied. This is very much confirmed by the value of the standard deviation of the card and in many cases is more than 60% of the average value.

**Figure 5:**
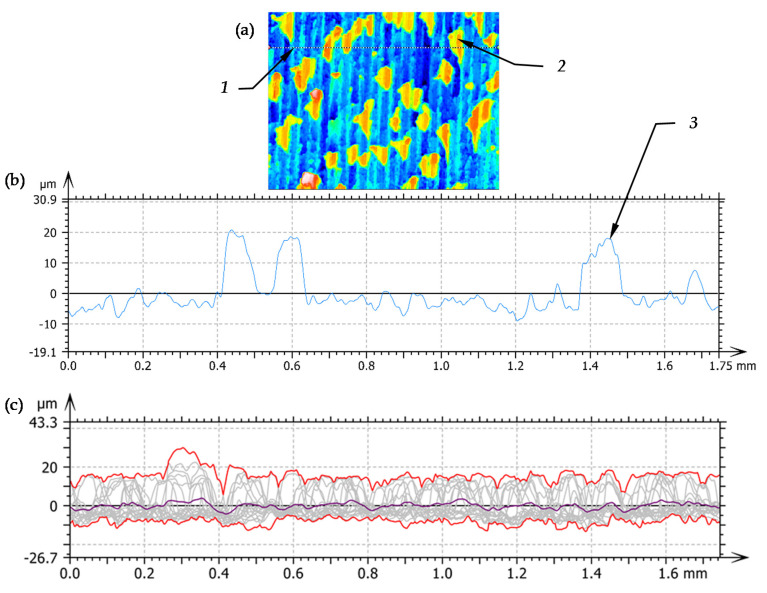
Extracted profile of surface after turning: (**a**) front view of turned surface, (**b**) profile of turned surface, 1—line of surface cross section, 2—surface peak, 3—profile surface peak, (**c**) the series of 19 west-east profiles extracted from the surface with upper/lower envelope and mean profile.

In order to compare the grain size to the substrate structure, a profile analysis was performed, where the surface profile was carried out by selected structure grains. Figure 5a,b shows the location of the profile in relation to the existing grains and the obtained profile. Additionally the series of 19 west-east profiles were extracted from the surface with upper/lower envelope and mean profile (Figure 5c) and statistical values of selected parameters of P-profile were included in Table 7.

On the presented profile you can see the structure of unevenness of the floor, the height of which does not exceed 10 µm. The harder grains of the structure create hills with an additional height of 20 µm protruding above the basic structure profile. The obtained profile was characterized by the parameters for the unfiltered profile, of which values are shown in Table 7. Analysing the parameters, it is possible to notice that single grains do not significantly affect the value of the average parameter, which is parameter Pa.

**Figure 6:**
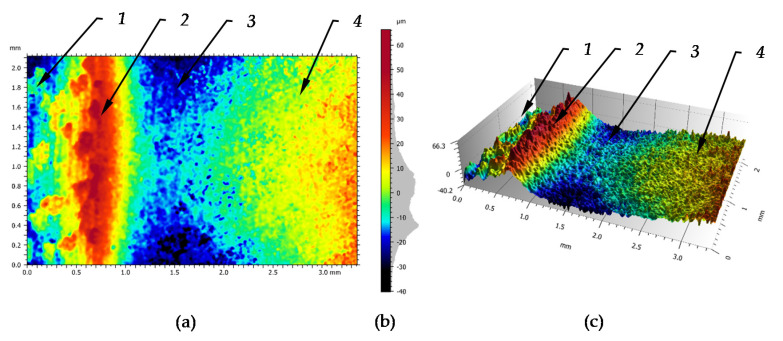
Surface deformation structure caused by the burnishing tool with visible effect of jumping wave: (**a**)—front view, (**b**)—scale of height and the ordinate histogram, (**c**)—3D view, 1—surface structure after turning, 2—jumping wave pushing by the tool, 3—surface structure under burnishing tool, 4—surface structure after burnishing.

The prepared surface was then subjected to the process of burnishing. The burnishing parameters are shown in Table 2. Figure 6 shows the structure of the surface occurring in the burnishing zone. The obtained structure is disturbed by elastic deformations of the material occurring in this zone, but these are small changes and their influence has been omitted in the study. This structure was obtained by stopping the kneading process and moving the tool away. In this Figure the zone of the surface structure can be specified before burnishing slightly deformed by the wave forming in front of the tool, this structure is marked as a 1. The area of wave crest as a zone of maximum deformation of the processed material which is formed before the burnishing tool is marked 2. The maximum tool pressure zone is marked as 3 and the area of the surface structure after burnishing is marked as 4.

Table 8 shows the surface parameters of the wave shown in Figure 6. These data are not generally the utility value, since such forms of processing usually are not left on the workpiece. However, for cognitive reasons, they are quite interesting from the point of view of the characteristics of this formation. E.g., parameter Sz informs about the total height of the forming wave, parameters Sp and Sv represent heights of hills to pits etc.

**Figure 7:**
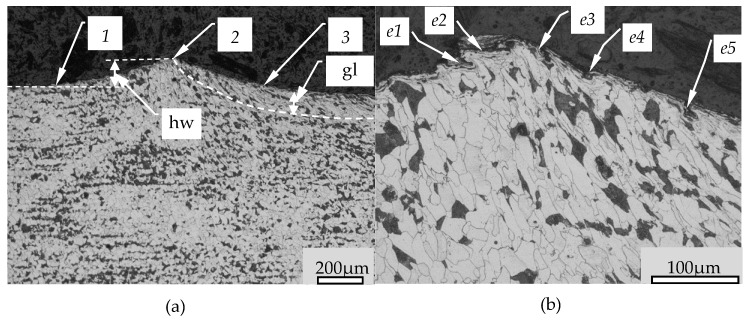
Jumping wave zone cross section: (**a**)–cross section from the jumping wave area (magnification 50×), (**b**)—top of jumping wave (magnification 200×), 1—surface structure after turning, 2—Top of Jumping wave, 3—structure under burnishing tool, e1–e5—subsequent changes in the structure of the surface flaws, *gl*—thickness of deformed layer, *hw*—height of jumping wave.

The Figure 7 presents the cross section of the “Jumping wave” before burnishing tool. In this Figure, the item 1 shows the surface before pressing, the item 2 crest of the wave and the item 3 presents the surface of influence of the burnishing tool. The pressure of the burnishing tool raises the processing material on the height indicated in Figure 7 by the item *hw*. Underneath the surface of the burnishing tool there is also a deformed zone marked *gl*. Significant deformation of the material structure can be seen in this zone. The wave in front of the tool is formed by the material rising in front of the tool. Even though in this structure material is pushed in over surface, however, the grains structure in the jumping wave does show slight differences form the core while the slight orientation of the grains towards the flow of the material can be observed. In the wave the rising is differentiated, which causes shear stress to occur between the different material zones. Shear stress causes slippages between zones in the generated wave. The displacement of the material layers in the wave causes creating additional surface irregularities in front of the burnishing tool. In Figure 7b are seen irregularities which occur when the layers of material slip in the wave and then move underneath the kneading tool where they are further deformed. Such a deformation scheme introduced additional defects (flaws) to the surface. The subsequent elements of the process of flaws creation is marked in Figure 7b as items e1–e5. In this Figure it can also be seen that the stresses that causes the wave of material creation also induced a change in the dominant direction of the grain arrangement in the plastic deformation zone. In this process, the workpiece material first flows upwards creating a wave and then is compressed by the burnishing tool, creating a zone of directional grain orientation. The movement of the material at different depths below the surface varies slightly.

The displacement of material on the surface of the processing element can be described in a form of point which is placed in the surface and its movement is specified relative to the workpiece. In this case it is first lifted by the wave in a vertical direction, then pressed by the burnishing tool, and returns to the surface level, but the path of return is different. This path is a diagonal curve of which direction corresponds with the feed, and in the last phase of the burnishing process there is a slight move back.

**Figure 8:**
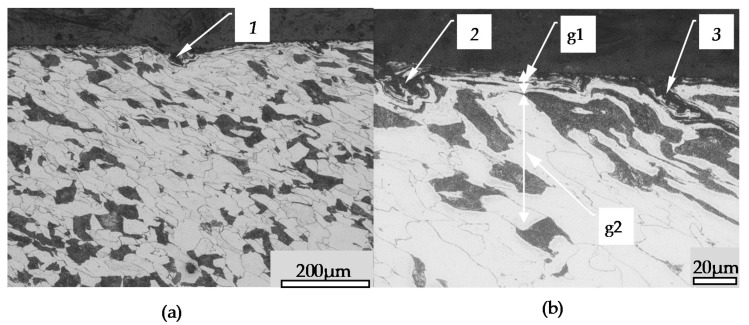
Surface flaws structure after the burnishing process: (**a**)—common view (magnification 100×), (**b**)—magnification of the surface flaws (magnification 500×), 1–3—surface flaws, g1—zone thickness of grain crushing and deformation, g2—zone thickness of the grain orientation.

Figure 8 shows the surface after burnishing. In this Figure item 1 shows the surface defect caused by the material slipping in the wave. Figure 8b shows an enlargement of the surface zone in which specific grain formations of the material marked as 2 and 3 are visible, as well as crushing zones g1 and g2 grain orientation which are visible. Very distorted and fragmented grains of material can be seen in zone g1. In zone g2, however, the grains are only slightly deformed and oriented by the tool.

**Figure 9:**
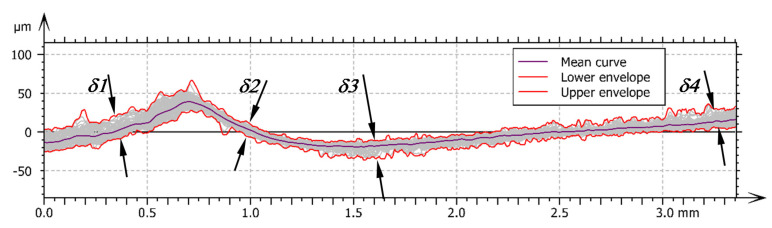
The sum of profiles of jumping wave structure: *δ1*—the thickness of the roughness structure after turning, *δ2*—the thickness of the roughness structure in front of the burnishing tool, *δ3*—the thickness of the roughness structure in bottom of the burnishing tool, *δ4*—the thickness of the roughness structure after burnishing.

Figure 9 shows a set of the profiles of the working section of the burnishing process. The thicknesses of the roughness zone are different in each segment of the burnishing process section. In the first segment is the wave which increases the initial roughness by the phenomena which occurs in the wave. In this segment the single irregularities are visible which were created by the slipping of burnishing material in the wave. The structure thickness in this segment is marked *δ1*. In the second segment of the burnishing process may be observed the roughness zone under the tool. This is the zone of direct action of the tool which the tool presses raised material against the surface of the workpiece. The roughness zone is smallest in this segment and is marked *δ2*. In segment three, the map surface is outside of the working area of the tool but is still in the suppress of waviness zone by the tool. The small range of reverse flow of material occurrence in this place causes the roughness zone to increase which is marked *δ3*. In the fourth zone, the surface is outside the tool; it is marked *δ4*. The mainly difference from zone 3 is the change in the roughness structure, i.e., from a predominantly valleys structure to a predominantly peaks structure, which may be due to the reverse flow of material described above.

The Table 9 present parameters surface irregularities were carried out based on the unfiltered profile. The statistical characteristics of the variability of these parameters are important data to determine the stability of the burnishing process.

From a practical point of view, the surface obtained as a result of the application of a given process is important in the burnishing process. For this purpose, a fragment area was separated from the above presented area of the sample surface and was analysed.

**Figure 10:**
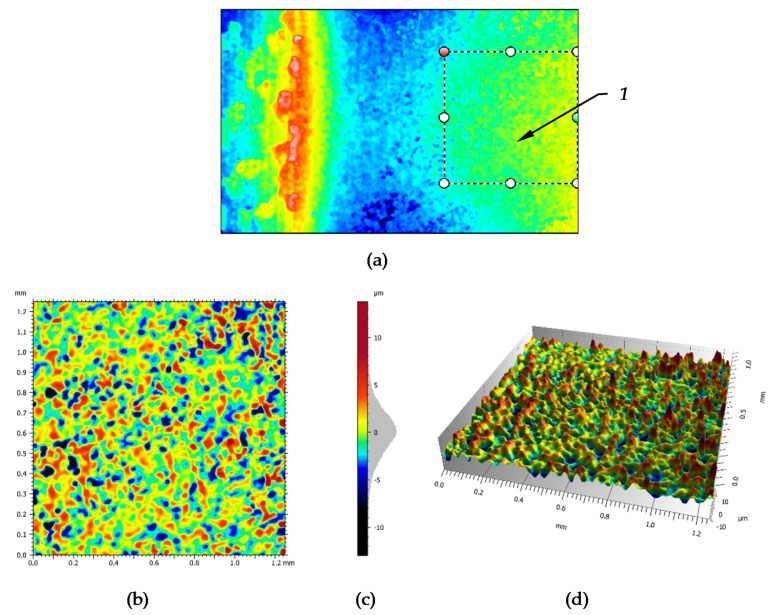
Surface structure occurs in the finishing segment of the burnishing zone: (**a**)—view of burnishing zone with the signed extraction area, (**b**)—front view of extracted area, (**c**)—scale of height and the ordinate histogram, (**d**)—3D view of extracted area.

The fragment area was separated from the contact zone of the conical part of the burnishing tool designed to decreasing surface waviness; the place is shown in Figure 10a. The enlargements of this fragment are shown in Figure 10b,d. In Figure 10c it can be seen that the ordinate distribution of the profile is basically symmetrical. Surface roughness parameters of this fragment are shown in Table 10.

**Figure 11:**
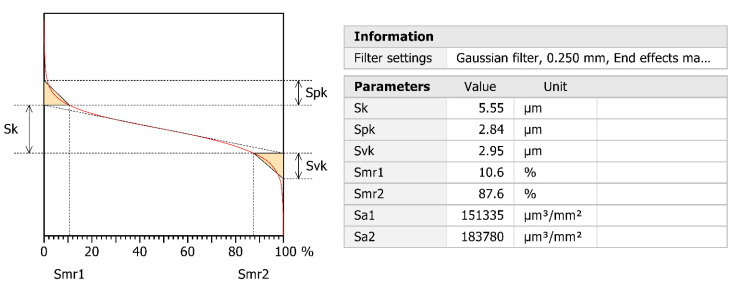
3D material ratio curve and its parameters for the extracted area surface.

From the point of view of utility of the surface, the parameters of material ratio curve are also important, which are presented in Figure 11. The curve presented is symmetrical where the values of reduced peaks and valleys have levelled values. Its relevant parameters are listed in the Table in Figure 11.

**Figure 12:**
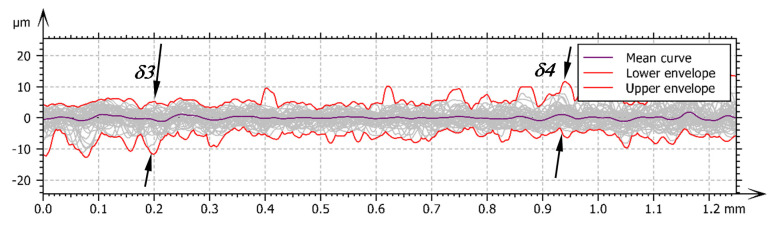
The sum of profiles of finishing segment of the burnishing zone: *δ3*—the thickness of the roughness structure in bottom of the burnishing tool, *δ4*—the thickness of the roughness structure after burnishing.

Figure 12 shows the assembly of profiles of the separated area. What may be seen is that in the zone of *δ3* markings, the surface is dominated by pits, while in the zone *δ4* additional peaks appear. This phenomenon may be caused by a slight flow of materials in the surface layer in the direction opposite to the feed vector in the burnishing process. Table 11 shows the statistical approach of the parameters of the surface profiles which are composed in the roughness zone visible in the Figure 12.

Analysing the data in Table 11, it can be seen that the mean values of the measured parameters are slightly different from the surface parameters determined by the S parameter. This applies to both amplitude and material ratio parameters. By the results of analysis of the standard deviation and the range of the findings, it can be concluded that extreme values of the parameters are relatively rare.

**Figure 13:**
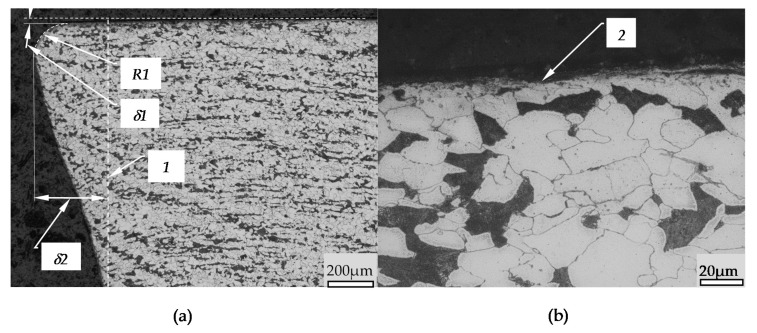
The cross-section of the shaft face surface: (**a**)—cross-section of the outflow which formed on the face surface from the jumping wave before the burnishing tool (magnification 50×), (**b**)—the cross-section of the surface boundary of the out flow (magnification 500×), 1—the outline of the face, 2—depression on the surface, R1—rounding radius of the face out flow, *δ1*—the lowered surface value in the facing zone, *δ2*—value of the distance of the out flow on the face surface.

Figure 13 presents an outline of the frontal surface, which was sucked out by moving the waves beyond the boundary of the burnished surface. The amount of pushed out material given to the frontal surface boundary varies with the distance from the processed surface. The shape of the obtained material structure indicates that the stresses above the yield point occur at a much greater depth. The grain structure of the stuffed material does not differ significantly from the core. The pressure of the rollers also caused a decrease in the crumpled surface in the frontal area. It can be seen that the maximum value of the displaced material occurs at a depth of *δ3*. The defects are on the surface in the form of grains strongly deformed, marked 2.

## 4. Conclusions

The phenomena occurring during wave formation in front of the burnishing tool were experimentally investigated. The occurring changes in the geometric texture of the surface are described by means of 2 and 3D parameters, while changes in the texture of the material are presented by means of micrographs of polished and etched samples.

The most important results of the experimental work can be summarized as follows:

- The shape of the outflow of the material wave beyond the front surface indicates that the stresses above the yield point during burnishing may occur at a much greater depth than would be apparent from the plastic deformation exists in the structure of the workpiece material.
- During the burnishing process the value of the surface roughness under the central point of the tool (the observation was made after moving out the tool from the surface) is lower in relation to the value of the surface roughness remaining after the pass of the tool. This may be the result of the reverse flow of material during the burnishing process (i.e., the flow direction is opposite to the feed) which causes an increase of the surface irregularities behind the tool.
- When material is pushed out in the wave, shear stresses occur that cause slippage of the material in the wave area which results the additional defects on the surface to be burnished. The flaws formed on the surface due to slips are not fully removed by the burnishing process and additional structures remain on the surface as residues of the material slips occurs in the wave before the burnishing tool.
- The created wave on the surface displaced in front of the tool causes the material in the surface layer to move in a vertical and horizontal direction. This can be seen from the specific orientation of the material structures in the surface layer. In the case when these displacements are too large the material can lose its cohesiveness and subsequently delaminate, what may lead to surface flaking.

Further research work will be focused on the investigation of the wave formation phenomena in the burnishing processes, realized under differentiated machining conditions.

## Figures and Tables

**Table 1:** Material data.

EN	C [wt.%]	Mn [wt.%]	Si [wt.%]	P [wt.%]	S [wt.%]	Cr [wt.%]	Ni [wt.%]	Al [wt.%]	Cu [wt.%]	Nb [wt.%]
1.0562	0.14	1.36	0.17	0.018	0.009	0.076	0.039	0.033	0.095	0.023
Parameter		Hardness (in a soft state)		Tensile strength (Rm)				The yield strength (Re)		
Unit		HB (Brinell Scale)	HRC (Rockwell scale)	MPa				MPa		
Value		220	<20	490–630				335		

**Table 2:** Processing parameters.

No of Sample	Diameter before Burnishing (after Turning) [mm]	Cutting Speed *Vc* [m/min]	Feed of Turning *ft* [mm/rev]	Diameter after Burnishing [mm]	Burnishing Speed *Vn* [m/min]	Feed of Burnishing *fn* [mm/rev]	Force *F* [N]
1	97.00 ± 0.02	61	0.3	96.92 ± 0.02	30.5	0.2	800
2	96.07 ± 0.02	61	0.3	96.00 ± 0.02	30.5	0.2	800
3	96.80 ± 0.02	61	0.3	96.70 ± 0.02	30.5	0.2	800

**Table 3:** 3-D roughness results acc. ISO 25178 [47] for surface after turning.

Height Parameters	Value	Unit	Description
Sq	7.53	µm	Root-mean-square height
Ssk	1.12	-	Skewness
Sku	3.09	-	Kurtosis
Sp	29.0	µm	Maximum peak height
Sv	12.9	µm	Maximum pit height
Sz	41.8	µm	Maximum height
Sa	6.06	µm	Arithmetic mean height

**Table 4:** Islands statistic for surface after turning.

Number of Islands–45, Threshold = 21.3 µm			
Parameters	Unit	Mean Value	Std. dev.
Projected area	mm^2^	0.00964	0.00822
Perimeter	µm	457	310
Equivalent diameter	µm	95.2	56.7
Mean diameter	µm	88.7	53.9
Min diameter	µm	56.4	33.3
Max diameter	µm	142	93.6
Min diameter angle	deg.	5.30	43.82
Max diameter angle	deg.	−16.1	64.41
Form factor	-	0.531	0.175
Aspect ratio	-	2.54	0.89
Roundness	-	0.481	0.149
Compactness	-	0.685	0.107
Orientation	deg.	70.0	38.8
Volume	µm^3^	51,496	50,492
Max height	µm	8.49	3.93
Height/Area ratio	µm/mm^2^	6025	9671
Mean height	µm	4.22	2.02

**Table 5:** Statistics over all grains—Binarized image after thresholding (21.3 µm).

Global Information	Value
Number of grains	47
Total area occupied by the grains	0.434 mm^2^ (19.0%)
Density of grains	20.5 Grains/mm^2^

**Table 6:** Grain statistic of the surface after turning.

Grain Parameters	Unit	Mean	Std. Dev.
Area	mm^2^	0.00924	0.00828
Perimeter	µm	440	317
Equivalent diameter	µm	91.3	58.5
Mean diameter	µm	84.9	55.5
Min diameter	µm	54.0	34.4
Max diameter	µm	136	95.7
Min diameter angle	deg.	4.13	42.1
Max diameter angle	deg.	−15.1	64.5
Form factor	-	0.529	0.170
Aspect ratio	-	2.63	0.961
Roundness	-	0.470	0.146
Compactness	-	0.677	0.106
Orientation	deg.	73.6	41.3

**Table 7:** Geometric structure parameters of unfiltered profile and series of 19 profiles for the turning surface acc. ISO 4287 [48].

**Amplitude Parameters** **Primary Profile**	**Unit**	**Value** **-**		**Context**	**Description**
Pp	µm	20.7			Maximum peak height of the raw profile.
Pv	µm	9.01			Maximum valley depth of the raw profile.
Pz	µm	29.7			Maximum height of the raw profile.
Pc	µm	13.6		ISO 4287 w/o amendment 2	Mean height of the raw profile elements.
Pt	µm	29.7			Total height of raw profile.
Pa	µm	4.92			Arithmetic Mean Deviation of the raw profile.
Pq	µm	6.88			Root-mean-square (RMS) Deviation of the raw profile.
Psk	-	1.64			Skewness of the raw profile.
Pku	-	4.63			Kurtosis of the raw profile.
The data for the series of 19 west-east profiles					
**Amplitude Parameters** **Primary Profile**	**Unit**	**Mean Value**	**Std. dev.**	**Context**	**Description**
Pp	µm	18.36	3.35	ISO 4287 w/o amendment 2	Maximum peak height of the raw profile.
Pv	µm	10.59	1.54		Maximum valley depth of the raw profile.
Pz	µm	28.95	2.82		Maximum height of the raw profile.
Pc	µm	18.32	4.00		Mean height of the raw profile elements.
Pt	µm	28.95	2.82		Total height of raw profile.
Pa	µm	5.99	1.09		Arithmetic Mean Deviation of the raw profile.
Pq	µm	7.40	0.84		Root-mean-square (RMS) Deviation of the raw profile.
Psk	-	1.11	0.48		Skewness of the raw profile.
Pku	-	3.25	1.58		Kurtosis of the raw profile.

**Table 8:** Results of 3-D Roughness parameters for the jumping wave zone acc. ISO 25178 [47].

Height Parameters	Value	Unit	Description
Sq	15.4	µm	Root-mean-square height
Ssk	0.696	-	Skewness
Sku	3.55	-	Kurtosis
Sp	66.3	µm	Maximum peak height
Sv	40.2	µm	Maximum pit height
Sz	107	µm	Maximum height
Sa	12.2	µm	Arithmetic mean height
Functional Parameters (Stratified surfaces) (used Gaussian filter, 0.25 mm)			
Sk	6.53	µm	Core roughness depth
Spk	4.69	µm	Reduced summit height
Svk	4.24	µm	Reduced valley depth
Smr1	11.7	%	Upper bearing area
Smr2	87.7	%	Lower bearing area
Spq	2.97		Plateau root-mean-square roughness
Svq	5.57		Valley root-mean-square roughness
Smq	94.4		Material ratio at plateau-to-valley transition

**Table 9:** Geometric structure parameters of unfiltered profile for the jumping wave acc. ISO 4287 [48].

Amplitude Parameters—Primary Profile	Unit	Mean	Std. Dev.	Min	Max	Lower Quartile	Upper Quartile	Median
Pp	µm	44.0	6.46	32.7	66.6	38.2	48.6	43.2
Pv	µm	25.6	4.38	20.2	36.3	22.2	28.2	23.8
Pz	µm	69.7	7.75	55.4	90.6	64.1	73.5	69.0
Pc (ISO 4287 w/o amendment 2)	µm	36.1	15.7	18.7	86.7	25.7	40.1	30.3
Pt	µm	69.7	7.75	55.4	90.6	64.1	73.5	69.0
Pa	µm	11.9	0.928	10.3	14.1	11.2	12.5	11.8
Pq	µm	15.0	1.03	13.2	17.8	14.0	15.8	15.0
Psk	-	0.788	0.257	0.222	1.38	0.630	0.991	0.753
Pku	-	3.41	0.686	2.12	5.59	2.95	3.87	3.33

**Table 10:** Results of 3-D Roughness parameters for the extracted area acc. ISO 25178 [47].

Height Parameters	Value	Unit	Description
Sq	2.62	µm	Root-mean-square height
Ssk	−0.0402	-	Skewness
Sku	4.59	-	Kurtosis
Sp	13.8	µm	Maximum peak height
Sv	13.0	µm	Maximum pit height
Sz	26.7	µm	Maximum height
Sa	1.99	µm	Arithmetic mean height

**Table 11:** The results of measurement roughness parameters from the profiles with the finishing segment of the burnishing zone acc. ISO 4287 [48].

Amplitude Parameters Roughness Profile	Unit	Context	Description	Mean	Std. Dev.	Min	Max
Rp	µm	Gaussian filter, 0.25 mm, End effects managed	Maximum peak height of the roughness profile.	3.97	0.748	2.74	5.78
Rv	µm	Gaussian filter, 0.25 mm, End effects managed	Maximum valley depth of the roughness profile.	4.01	0.743	2.47	6.03
Rz	µm	Gaussian filter, 0.25 mm, End effects managed	Maximum Height of roughness profile.	7.98	1.31	5.31	10.6
Rc	µm	Gaussian filter, 0.25 mm, End effects managed, ISO 4287 w/o amendment 2	Mean height of the roughness profile elements.	5.22	0.823	3.14	7.11
Rt	µm	Gaussian filter, 0.25 mm, End effects managed	Total height of roughness profile.	12.1	2.93	6.92	18.2
Ra	µm	Gaussian filter, 0.25 mm, End effects managed	Arithmetic mean deviation of the roughness profile.	1.63	0.247	1.02	2.11
Rq	µm	Gaussian filter, 0.25 mm, End effects managed	Root-mean-square (RMS) deviation of the roughness profile.	2.02	0.316	1.28	2.62
Rsk	-	Gaussian filter, 0.25 mm, End effects managed	Skewness of the roughness profile.	-0.011	0.250	-0.48	0.61
Rku	-	Gaussian filter, 0.25 mm, End effects managed	Kurtosis of the roughness profile.	2.71	0.338	2.18	3.70
Material ratio parameters—Roughness profile							
Rmr	%	c = 1 µm under the highest peak, Gaussian filter, 0.25 mm	Relative Material Ratio of the roughness profile.	3.17	2.45	0.77	13.9
Rdc	µm	p = 20%, q = 80%, Gaussian filter, 0.25 mm	Roughness profile Section Height difference	3.16	0.505	1.95	4.23
ISO 13565 [49], ISO 13565-2 [50]							
Rk	µm	Double Gaussian filter, 0.25 mm, End effects managed	Kernel roughness depth.	4.79	0.797	2.91	6.40
Rpk	µm	Double Gaussian filter, 0.25 mm, End effects managed	Reduced peak height.	2.09	0.893	0.95	4.89
Rvk	µm	Double Gaussian filter, 0.25 mm, End effects managed	Reduced valley depth.	3.07	0.925	1.30	5.45
Mr1	%	Double Gaussian filter, 0.25 mm, End effects managed	Upper material ratio.	9.81	3.08	4.11	17.2
Mr2	%	Double Gaussian filter, 0.25 mm, End effects managed	Lower material ratio.	85.1	4.03	73.4	95.0
A1	µm^2^/ mm	Double Gaussian filter, 0.25 mm, End effects managed	Upper area.	105	58.6	28.3	276
A2	µm^2^/ mm	Double Gaussian filter, 0.25 mm, End effects managed	Lower area.	236	113	43.3	626
Rpk*	µm	Double Gaussian filter, 0.25 mm, End effects managed	Peak height.	3.08	1.68	1.01	7.61
Rvk*	µm	Double Gaussian filter, 0.25 mm, End effects managed	Valley depth.	4.78	1.99	1.84	11.2

Rpk*: Peak height; Rvk*: Valley depth.

## Data Availability

Data are contained within the article.
